# Bioethical committees and data protection issues in Poland

**DOI:** 10.1186/1476-069X-7-S1-S4

**Published:** 2008-06-05

**Authors:** Danuta Ligocka

**Affiliations:** 1 Nofer Institute of Occupational Medicine, 8 Teresy St., 91-348 Lodz, Poland

## Abstract

In Poland there are only Regional Bioethical Committees. Unlike most EU countries Poland has no coordinating centre on bioethics for human research. However, the Ministry of Health and Welfare has established a Bioethics Appeals Committee.

The functioning of the Bioethical Committees in Poland is regulated in detail by the Regulation of the Ministry of Health and Welfare of 1999. All regulations comply with important guidelines such as: the Helsinki Declaration, The Rules of Good Clinical Practice, EU Directives and legal regulations binding in Poland, mainly the Act of the Medical Doctor Profession and the Dentist Profession, as well as the Act of Pharmaceutical Law.

In the framework of the Human Biomonitoring Programme, the application for bioethical evaluation will be submitted to the Bioethical Committee at the Nofer Institute of Occupational Medicine in Lodz.

The data protection legislation in Poland according to the Act of the Protection of Personal Data of 29^th ^of August 1997 with latest amendments fulfils EU regulations. The Act also contains detailed provisions regarding the duties of the Inspector General for Data Protection. The paper presents data on the activities of the Bureau of the Inspector General for Personal Data Protection in 2005, 2006 and 2007.

## Bioethical Committees in Poland

In Poland, a number of bodies responsible for dealing with ethical issues of research have been established. There are twenty Regional Ethics Committees for Animal Research coordinated by the National Ethics Committee for Animal Research.

Unlike other countries, Poland does not have a coordinating national centre for Bioethical Committees in human research. There are 54 independent Bioethical Committees. The topics of applications submitted for ethical evaluation to the Bioethical Committees vary according to the research area of the institutions applying.

The Bioethical Committees are independent collegial bodies. The aim of their activity is to guarantee the rights, safety and welfare of the participants in medical research. The Committee safeguards the respect and dignity of a human being with higher priority than the scientific aims of the research.

The functioning of Bioethical Committees in Poland is regulated by the Act of the Medical Doctor Profession and the Dentist Profession of 5^th ^of December 1996 (with later amendments) [1], Act of Pharmaceutical Law of 6^th ^of October 2001 [2], Regulation of the Minister of Health and Welfare of 11^th ^of May 1999 [3]. All regulations comply with the most important international guidelines formulated in: the Helsinki Declaration, the International Ethical Guidelines for Bioethical Research Involving Human Subjects (issued by the Council for International Organization of Medical Sciences), the guidelines of Good Clinical Practice, as well as legal regulations binding in Poland.

There are 54 Bioethical Committees in Poland and their activities depend on the main tasks of the institutions. The 24 appointed at the Regional Medical Councils mainly evaluate projects related to a new drugs pre-registration process, the 19 Committees at the Research Medical Institutes approve research projects concerning new therapy, diagnostics and monitoring methods, while those located at Medical Universities cover the whole spectrum: pharmacology, diagnostics, therapy research and pre-registration drug evaluation.

The Ministry of Health and Welfare supports the European Human Biomonitoring Programme, and Prof. Marek Jakubowski, the Nofer Institute of Occupational Medicine is the national work package leader. Within the framework of the pilot project, the application for bioethical evaluation will be submitted to the Bioethical Committee at the Nofer Institute of Occupational Medicine in Lodz.

## The Nofer Institute of Occupational Medicine (NIOM)

The Nofer Institute of Occupational Medicine (NIOM), Lodz, Poland, was founded in 1954. During a period of more than five decades the institute has evolved from a small unit at the Medical University of Lodz into an independent complex research and development centre with activities covering various areas of occupational and environmental health. The activities of the Nofer Institute have gained considerable significance at both national and international levels. During the socioeconomic transition of the Central and Eastern European countries, the Institute has actively participated in the process of restructuring the occupational health services and prepared drafts of new legal acts on Occupational Health Service, chemical safety, social insurance system, etc [6].

The major research areas of the Nofer Institute of Occupational Medicine are:

• occupational medicine, environmental health, public health, occupational hygiene, radiation protection (ionising and non-ionising radiation)

• occupational pathology, prevention of occupational and work-related diseases with special regard to occupational allergy, diagnostics and treatment of acute poisonings

• toxicology, including toxicometry, toxicity assessment of chemicals and methodology of exposure assessment of chemical hazards in work environment

• epidemiology of occupational diseases and other health effects of exposure to physical, chemical and psychosocial hazards

• assessment of health risks from environmental exposure to hazardous agents

• physiology, psychology, sociology of work and ergonomics

• health promotion in the workplace

• management and economics of occupational health services

• health-based criteria of vocational selection and capability for work

The Bioethical Committee at the Nofer Institute of Occupational Medicine is appointed by the Internal Ordinance of the Director of NIOM for the period of three years and performs its functions until a new committee is appointed for the next term. Of its 13 members eight are medical doctors chosen among the researchers at NIOM according to their professional competence and experience in the field of research, while five are experts, independent of NIOM, with academic competence in theology, philosophy, pharmacy, law and health care service.

The decisions of the Committee concerning expressing opinion about the research projects are taken in the form of the resolution made by a secret ballot and only the votes for (a yes) or against (a no) the project can be approved [7].

The major topics of the applications submitted for ethical evaluation within the Bioethical Committees at NIOM are: a) development of new diagnostic methods for occupational diseases, b) development and validation of new biomarkers of exposure to chemicals both in the occupational setting and under experimental conditions (human volunteer studies), c) validation of usefulness of some biomarkers of effect (epidemiological studies), d) assessment of health risks from environmental exposure to hazardous agents.

Some examples of applications recently submitted to the Bioethical Committee at NIOM are:

"Night-shift work melatonin metabolism and breast cancer risk factors in nurses", "Dietary exposure to PAHs and DNA damage", "Usefulness of molecular biology techniques for oxidative stress assessment in breast cancer patients", Environmental exposure assessment of populations with different level of Methyl mercury intake".

The table on the Ethics Committees in Poland developed by ESBIO is presented in Additional file 1.

## Personal data protection

Provisions for regulating personal data processing were introduced in Polish legislation by the Act of Personal Data Protection of 29^th ^of August 1997 (with later amendments) [8]. This Act fulfils the Directive 95/46/EC of the European Parliament and of the Council.

According to article 12 of the Act the duties of the Inspector General are: 1) to ensure the compliance of data processing with the provisions of the act of protection of personal data; 2) to issue administrative decisions and consider complaints with respect to the enforcement of the regulations on the protection of personal data; 3) to maintain the register of data filing systems and provide information of the registered data filing systems; 4) to issue opinions on draft laws and regulations with respect to the protection of personal data; 5) to initiate and undertake activities aimed at more efficient protection of personal data; 6) to participate in the work of international organisations and institutions involved in personal data protection [9].

The Act of Personal Data Protection states that informed consent must always be obtained from the data subject prior to processing of his or her personal data. Sensitive information may be processed upon written consent given by the data subject. Sensitive information is information on racial or ethnic origin, political opinions, religious or philosophical beliefs, religious, political party or trade-union membership, health records, genetic code, addictions or sexual life.

The Inspector General for Personal Data Protection participates in the work of international organizations [10]:

• co-operates with international organisations involved in the protection of data,

• participates as an observer in the Working Party established on the basis of Article 29 of the Directive 95/46/EC of the European Parliament and of the Council of 24^th ^of October 1995 of the protection of individuals with regard to the processing of personal data and on the free movement of such data,

• appoints members of the Joint Supervisory Body of Europol (JSB Europol) and their alternates and nominates the candidates for a member of the Appeals Committee and his/her alternate,

• participates as an observer in the meetings of Joint Supervisory Authority of Schengen (JSA Schengen),

• participates as an observer in the meetings of Joint Supervisory Authority Customs (JSA Customs),

• co-operates with other countries' commissioners for data protection.

The Table on data protection in Poland developed by ESBIO is presented in Additional file 2.

The main tasks of the IGPD are registration of files, inquiries concerning the Act and its interpretation, and complaints. The last months of the year appear more active with most data submitted for registration and inquiries.

As shown in a Table 1 there are about. 5000 files submitted for registration per year while around 3000 per year are registered. The number of inquiries concerning the interpretation of the Act has decreased with 50% within the last 2 years.

**Table 1:** Statistics of Activity of the Bureau of the Inspector General for Personal Data Protection – according to [10]

			YEAR		
			
Type of Activity			2005	2006	2007
Sent to the Inspector General	Inquiries concerning the binding Act and its interpretation		2821	2228	1298
	Complaints		979	712	796
	Bills submitted for opinion		327	265	348
Inspections conducted by the Inspector General			119	108	167
Decisions	Jurisprudence, Legislation and Complaints Department *			38	280
	Legal Department		6	7	-
	Inspection Department		98	101	130
	Registry Department	Refusal of registration	123	215	173
		Discontinuation of registration proceedings	78	50	40
		Removal from the nationwide registry of personal data filling system	181	533	148
	Complaints Department		336	319	-
Total			822	1263	771
Notification of committed offences			52	15	18
Files	Submitted for registration		5344	4743	4850
	Registered		3175	3610	2598

The possibility of removal from the nationwide registry of personal data filing systems was established as a result of the amendment to the Act on the Personal protection Data (Article 44, active 1 May 2004).

* – The Legal Department and the Complaints Department ceased to exist as of 10 November 2006. As a result of organizational changes, that is the combination of these two departments, the Jurisprudence, Legislation and Complaints Department was established.

The processing of personal data in violation of the Act may result in a ban of processing the data. Moreover, such unlawful processing is a crime, thus the management of the company which is not fulfilling all obligations may be subject to a fine, a limitation of liberty (such limitation may involve a prohibition on changing residence without the court's consent or an obligation to render social services for a period of up to one year), or imprisonment for up to three years [10].

## Conclusion

Polish regulations comply with most of the EU and international rules with respect to ethical issues and personal data protection. Poland does not have a Central Ethics Committee as in other EU member states. There are 54 independent Bioethical Committees in Poland which regards the respect for the dignity of a human being as superior to scientific aims of research.

## Competing interests

The authors declare that they have no competing interests.

**Figure 1:**
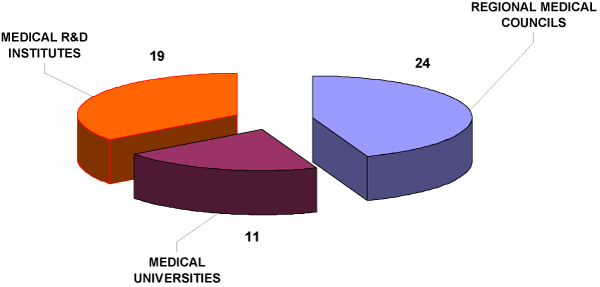
Bioethical Committees in Poland.

## Supplementary Material

Additional file 1 Overview: RECs in Poland Click here for file

Additional file 2 Overview: Data protection in Poland Click here for file
