# Supplementary material for: Bioethical committees and data protection issues in Poland
Source: Environ Health. 2008 Jun 5;7(Suppl 1):S4. doi: 10.1186/1476-069X-7-S1-S4 (PMC2423453; doi:10.1186/1476-069X-7-S1-S4)
Supplement: Additional file 1 — Overview: RECs in Poland [file 1476-069X-7-S1-S4-S1.doc]

ESBIO

Overview: RECs in Poland:

Introductory notes:

Poland does not have a Central Ethics Committee as in other EU member states. The work of RECs are thus conducted at a medical R&D Institution. This overview will focus on the Bioethical Commission based at the Nofer Institute of Occupational Medicine (NIOM).

|  | **Medical R&D Institute - NIOM** |
| --- | --- |
| **Type of REC** | Commission of Bioethics. |
| **Where situated?** | Bioethical Commission at the Nofer Institute of Occupational Medicine in Lodz |
| **Who applies to them?** | Researchers. |
| **Legal provisions** | The Commission of Bioethics issued on the basis of the Internal Regulation No 7/2004 of the Head of the Nofer Institute of Occupational Medicine in Lodz of 9^th^ August 2004.  The Bioethical Commission is based upon the Regulation of the Minister of Health and Social Care of May 11, 1999 on the detailed rules of appointing and financing as well as the mode of activity of bioethical commissions. Also the Act of medical doctor profession and the dentist profession of December 5, 1996 (Journal of Laws 1997, No 28, 152; last amendment Journal of Laws 2006, No 117, 790) |
| **Official opinion on Biomonitoring** | No opinion stated. |
| **Responsible/accountable to whom?** | Independent. |
| **Mission/tasks** | The Commission in its opinions is directed by ethical values and the purposefulness and feasibility of the research projects.  *Contribution to guaranteeing the rights, safety and welfare of subjects of clinical research. The Commission guards the respect for the dignity of a human being as the superior value to scientific goals of research. – that’s part of the act of medical doctor profession…* |
| **Approval or Advisory powers** | Approval. |
| **Not submitting research or failing to follow review**  **Which body, if not the REC itself takes this action, and how do they proceed?** | *In Article 27 of the Act of medical doctor profession ... there is additional information which I will explain later, after consultation.*  There are no legally defined penalties for failure to follow review, although it is assumed that medical professionals are legally and professionally responsible for their research.  In cases regarding doctors, it would either be the Medical Court of the local Medical chambers or the Supreme Medical Court of the main Medical Chamber. The courts would consider the doctors' adherence to the principles of the Code of Medical Ethics. They have the power to award various penalties and/or revoke a doctor’s licence for serious breaches of the Code. If there was a breach of the law, then the courts would deal with the case. *Source: Privireal.* |
| **Application procedure** | §2.  1. A person or other subject intending to conduct a medical experiment shall lodge an “Application for Opinion on a Medical Experiment Project” (hereinafter referred to as “the application”) with the Bioethical Commission.  2. The application shall include:   1. identification of the person or other subject intending to conduct a medical experiment, or, in the case of a multicentre experiment – also the names of all throughout the country where the experiment is to be conducted, 2. tile of project, its detailed description and a statement of reasons concerning its justification and feasibility, 3. name and surname, address and professional as well as scientific qualifications of the person in charge of medical experiment, 4. information on the terms of insurance for the persons who are to participate in the medical experiment, 5. information on the expected curative and cognitive benefits and possibly other expected advantages for persons subjected to the medical experiment.   3. The following shall be attached to the application:   1. description of the medical experiment 2. information designed for persons subjected to the medical experiment including the details of the aims and principles of conducting the medical experiment, the expected curative and other benefits for these persons and the risk related to being subjected to the experiment, 3. a sample of patient consent form for a person subjected to the medical experiment which should include, at least, statements concerning:  - voluntary consent to being subjected to the medical experiment after becoming acquainted with the information referred to in subparagraph 2, - confirmation of the possibility of asking the experiment leader questions and receiving answers to these questions, - obtaining information on a possibility of withdrawing from the medical experiment at every stage of it,  1. a sample of declaration of the acceptance of insurance terms, 2. a sample of statement made by the person subjected to the medical experiment in which he/she gives consent to processing the data related to his/her participation in the experiment by the person or other subject conducting the medical experiment   4. If a person who is unable to give his/her written consent, a minor or fully incapacitated person is to take part in the medical experiment, the sample of documents referred to in Article 3, subparagraphs 3-5 should allow for the confirmation of the data resulting from the circumstances specified in Article 25, subparagraphs 1, 2, and 4 of the Act of December 5, 1996, on the profession of physician (Jour. Of Law of 1997, No 28, item 152 with further amendments), hereinafter referred to as “the Act”. |
| **Is it required that the REC approve data from third party?** | There are no provisions regarding third party REC approval |
| **Provisions for not submitting completed projects?** | §3  If the lodged documents referred to in § 2, hereinafter “the experiment documentation”, are incomplete, the Biomedical Commission shall return them to the applicant to be completed. |
| **RECs position in regards to incentives** | No opinion stated. *However, there are some regulation of the Head of NIOM concerning on incentives* |
