# Supplementary material for: Bioethical committees and data protection issues in Poland
Source: Environ Health. 2008 Jun 5;7(Suppl 1):S4. doi: 10.1186/1476-069X-7-S1-S4 (PMC2423453; doi:10.1186/1476-069X-7-S1-S4)
Supplement: Additional file 2 — Overview: Data protection in Poland [file 1476-069X-7-S1-S4-S2.doc]

ESBIO

Overview: Data Protection in Poland:

| **Title of Data Protection Legislation** | Act on the Protection of Personal Data. Last amended in 2006. |
| --- | --- |
| **Content of the Data Protection Legislation**  **Personal Data**  **Inspector General for Personal Data Protection**  **Biobanks**  **Sensitive Information** | *Personal Data* is defined as:  Any information relating to an identified or identifiable natural person. Identifiable natural person is one who can be identified, directly or indirectly, in particular by reference to an identification number or to one or more factors specific to his/her physical, physiological, mental, economic, cultural or social identity. A piece of information shall not be regarded as identifying where the identification requires an unreasonable amount of time, cost and manpower.[[1]](#footnote-2)  The Act contains detailed provisions regarding the **duties** of the Inspector General:  **Article 12**: "The duties of General Inspector shall include in particular:  1) ensuring the compliance of data processing with the provisions of the act on the protection of personal data;  2) issuing administrative decisions and consideration of complaints with respect to the enforcement of the regulations on the protection of personal data;  3) keeping the register of data filing systems and providing information on the registered data filing systems;  4) issuing opinions on draft laws and regulations with respect to the protection of personal data;  5) initiating and undertaking activities aimed at more efficient protection of personal data;  6) participating in the work of international organisations and institutions involved in personal data protection".  **Article 14**: "To perform the duties referred to in Article 12.1 and 12.2 General Inspector or inspectors authorised by General Inspector shall enjoy the following powers, and in particular: 1) The power to enter, from 6 a.m. through 10 p.m., after presenting the adequate personal authorisation and service identity card, any premises where the registered data filing system is being kept and to perform necessary examination or other inspection activities to assess the compliance of the data processing activities with the Act;  2) The power to demand written or oral explanation and the power to summon and hear any person with regard to determining the actual state of things;  3) The power to demand presentation of documents and any data relating to the subject of the control;  4) The power to demand that any devices, data carriers, and automatic systems of data processing be submitted for the purpose of examination; 5) The power to order expert analysis and opinions to be prepared".  **Article 18:**  “Should the inspection reveal any breach of the provisions on the protection of personal data, General Inspector, on his own initiative or on request of the interested party, shall order the controller, by means of administrative decision, to restore the state compliant with the law, and in particular: 1) to eliminate any failure;  2) to complete, update, correct, disclose or keep confidential the personal data;  3) to apply additional measures protecting the personal data files;  4) to suspend the transmission of personal data to third countries;  5) to safeguard the data or to transfer them to other entities; or  6) to erase the personal data". *Source: Privireal.*  There is no reference to biobanking in the Data Act.  Sensitive information is information on racial or ethnic origin, political opinions, religious or philosophical beliefs, religious, party or trade-union membership, health records, genetic code, addictions or sexual life. |
| **Damages and criminal penalties**  **What are the penalties for data controllers if they breach the law?** | Chapter 8 of the Act determines that: Quote:  **Article 49**  “1. A person, who processes personal data in a data filing system where such processing is forbidden or where he/she is not authorised to carry out such processing, shall be liable to a fine, a partial restriction of freedom or a prison sentence of up to two years.  2. Where the offence mentioned in point 1 of this article relates to information on racial or ethnic origin, political opinions, religious or philosophical beliefs, religious, party or trade-union membership, health records, genetic code, addictions or sexual life, the person who processes the data shall be liable to a fine, a partial restriction of freedom or a prison sentence of up to three years.  **Article 50**  A person who, being the controller of a data filing system, stores personal data incompatibly with the intended purpose for which the system has been created, shall be liable to a fine, the penalty of restriction of liberty or deprivation of liberty up to one year.  **Article 51**  1. A person who, being the controller of a data filing system or being obliged to protect the personal data, discloses them or provides access to unauthorised persons, shall be liable to a fine, the penalty of restriction of liberty or deprivation of liberty up to two years.  2. In case of unintentional character of the above offence, the offender shall be liable to a fine, the penalty of restriction of liberty or deprivation of liberty up to one year.  **Article 52**  A person who, being the controller of a data filing system violates, whether intentionally or unintentionally, the obligation to protect the data against unauthorised takeover, damage or destruction, shall be liable to a fine, the penalty of restriction of liberty or deprivation of liberty up to one year.  **Article 53**  A person who, regardless of the obligation, fails to notify the data filing system for registration, shall be liable to a fine, the penalty of restriction of liberty or deprivation of liberty up to one year.  **Article 54**  A person who, being the controller, fails to inform the data subject of its rights or to provide him/her with the information which would enable that person to benefit from the provisions of this Act, shall be liable to a fine, partial restriction of freedom or prison sentence of up to one year.” |
| **Provisions on the processing of a national identification number or a general identifier, as per Article 8(7) of Directive 95/46/EC, if any** | Yes. The main provisions in this regard are contained in the Act on Census and Identification Documents 1974. |
| **Informed Consent** | The Act states that *consent* must always be obtained from the data subject prior to *processing* of his personal data. The obligation to obtain consent is extended to sensitive information. Sensitive information may not be processed unless explicit written consent has been given by the data subject.  Consent is not required prior to collection of personal data. However in most circumstances the data controller is obligated to notify the data subject that personal data has been collected from the data subject. Exceptions do exist where notification of the data subject is not required in case of any other polish law stating that notification is not required.  According to the Act consent may also be obtained for future processing of personal data, as long as the purpose of the processing is not changed. |
| **Rights of the data subject**   1. **Right and access to information** 2. **Right to request correction and erasure of personal data** | Chapter 4 of the Act provides detailed provisions regarding the rights of the data subject. In short the data subject is entitled to receive information upon request whether any data is being stored concerning the data subject. Furthermore the data subject is entitled to be notified of the identity of the data controller and also the purpose, scope and the means of any processing of the data stored aswell as possible disclosure to third parties.  **Article 35**  “1. Should the data subject prove that the personal data relating to him/her are not complete, they are outdated, untrue or collected with the violation of the Act, or in case they are no longer required for the purpose for which they have been collected, the controller shall be obliged, without undue delay, to amend, update, or correct the data, or to temporarily or permanently suspend the processing of the questioned data, or to have them erased from the filing system, unless the above refers to the personal data which shall be amended, updated or corrected pursuant to the principles determined by other laws.  2. Should the controller fail to fulfil the obligation referred to in paragraph 1 above, the data subject may apply to the Inspector General to issue a relevant order to the controller.  3. The controller shall be obliged to inform without undue delay other controllers, to whom he/she disclosed a data file, that some data have been updated or corrected.” |
| 1. **Processing of data** 2. **Transfer to third party** 3. **Security of Personal Data** | Chapter 3 of the Act. Concerning ***personal data*** the Act states:  **Article 23**  “1. The processing of data is permitted only if:  1) the data subject has given his/her consent, unless the processing consists in erasure of personal data,  2) processing is necessary for the purpose of exercise of rights and duties resulting from a legal provision,  3) processing is necessary for the performance of a contract to which the data subject is a party or in order to take steps at the request of the data subject prior to entering into a contract,  4) processing is necessary for the performance of tasks provided for by law and carried out in the public interest,  5) processing is necessary for the purpose of the legitimate interests pursued by the controllers or data recipients, provided that the processing does not violate the rights and freedoms of the data subject.  2. The consent referred to in paragraph 1, point 1 may also be applied to future data processing, on the condition that the purpose of the processing remains unchanged.  3. Should the processing of data be necessary to protect the vital interests of the data subject and the condition referred to in paragraph 1, point 1 cannot be fulfilled, the data may be processed without the consent of the data subject until such consent can be obtained.  4. The legitimate interests, referred to in paragraph 1, point 5 in particular, are considered to be:  1) direct marketing of own products or services provided by the controller,  2) vindication of claims resulting from economic activity.”  Concerning *sensitive personal data* the Act states:  **Article 27**  “1. The processing of personal data revealing racial or ethnic origin, political opinions, religious or philosophical beliefs, religious, party or trade-union membership, as well as the processing of data concerning health, genetic code, addictions or sex life and data relating to convictions, decisions on penalty, fines and other decisions issued in court or administrative proceedings shall be prohibited.  2. Processing of the data referred to in paragraph 1 above shall not constitute a breach of the Act where:  1) the data subject has given his/her written consent, unless the processing consists in erasure of personal data,  2) the specific provisions of other statute provide for the processing of such data without the data subject's consent and provide for adequate safeguards,  3) processing is necessary to protect the vital interests of the data subject or of another person where the data subject is physically or legally incapable of giving his/her consent until the establishing of a guardian or a curator,  4) processing is necessary for the purposes of carrying out the statutory objectives of churches and other religious unions, associations, foundations, and other non-profit-seeking organisations or institutions with a political, scientific, religious, philosophical, or trade-union aim and provided that the processing relates solely to the members of those organisations or institutions or to the persons who have a regular contact with them in connection with their activity and subject to providing appropriate safeguards of the processed data,  5) processing relates to the data necessary to pursue a legal claim,  6) processing is necessary for the purposes of carrying out the obligations of the controller with regard to employment of his/her employees and other persons, and the scope of processing is provided by the law,  7) processing is required for the purposes of preventive medicine, the provision of care or treatment, where the data are processed by a health professional subject involved in treatment, other health care services, or the management of health care services and subject to providing appropriate safeguards,  8) the processing relates to those data which were made publicly available by the data subject,  9) it is necessary to conduct scientific researches including preparations of a thesis required for graduating from university or receiving a degree; any results of scientific researches shall not be published in a way which allows identifying data subjects,  10) data processing is conducted by a party to exercise the rights and duties resulting from decisions issued in court or administrative proceedings.”  **Article 47**  “1. The transfer of personal data to a third country may take place only, if the country of destination ensures at least the same level of personal data protection in its territory as that in force in the territory of the Republic of Poland.  2. The provision of paragraph 1 above shall not apply to the transfer of personal data required by legal provisions or by the provisions of any ratified international agreement.  3. Nevertheless the controller may transfer the personal data to a third country provided that:  1) the data subject has given his/her written consent,  2) the transfer is necessary for the performance of a contract between the data subject and the controller or takes place in response to the data subject's request,  3) the transfer is necessary for the performance of a contract concluded in the interests of the data subject between the controller and another subject,  4) the transfer is necessary or required by reasons of public interests or for the establishment of legal claims,  5) the transfer is necessary in order to protect the vital interests of the data subject,  6) the transfer relates to data which are publicly available.  **Article 48**  In cases other than those referred to in Article 47 paragraph 2 and 3 the transfer of personal data to a third country which does not ensure at least the same level of personal data protection as that in force in the territory of the Republic of Poland, may take place subject to a prior consent of the Inspector General, provided that the controller ensures adequate safeguards with respect to the protection of privacy, rights and freedoms of the data subject.”  **Article 36**   1. The controller shall be obliged to implement technical and organisational measures to protect the personal data being processed, appropriate to the risks and category of data being protected, and in particular to protect data against their unauthorised disclosure, takeover by an unauthorised person, processing with the violation of the Act, any change, loss, damage or destruction. 2. The controller shall keep the documentation describing the way of data processing and measures referred to in paragraph 1. 3. The controller shall appoint an administrator of information security who supervises the compliance with security principles referred to in paragraph 1, unless the controller performs these activities by himself. |
| **Distinctive characteristics/features on Data Protection in Poland compared to other EU member states** |  |

1. Article 6 of the Act. [↑](#footnote-ref-2)
